# Respectful maternity care in the UK using a decolonial lens

**DOI:** 10.1007/s43545-022-00576-5

**Published:** 2022-12-04

**Authors:** Amali U. Lokugamage, Nathan Robinson, Sithira D. C. Pathberiya, Sarah Wong, Christine Douglass

**Affiliations:** 1 Institute of Women’s Health, UCL. 74 Huntley St, London, WC1E 6DE UK; 2 Present Address: UCL Medical School, University College London, 74 Huntley St, London, WC1E 6DE UK; 3 Present Address: Radius Law Solicitors, London, UK; 4 Present Address: HWU (EDI), Edinburgh, Scotland

**Keywords:** Obstetrics, Patient perspective, Decolonisation, Power imbalances, Health personnel, Paternalism, Respectful maternity care, Racism

## Abstract

Respectful maternity care (RMC) is part of a global movement addressing the previous absence of human rights in global safe maternal care guidance. RMC is grounded in kindness, compassion, dignity and respectful working conditions. The decolonisation movement in healthcare seeks to dismantle structural biases set up from a historically white, male, heteronormative Eurocentric medical system. This article applies a decolonising lens to the RMC agenda and examines barriers to its implementation in UK healthcare systems. Searches of peer-reviewed journals about decolonising maternity care in the UK revealed little. Drawing from wider information bases, we examine power imbalances constructed throughout a history of various colonial biases yet lingering in maternity care. The overarching findings of our analysis revealed 3 areas of focus: professional structures and institutional biases; power imbalances between types of staff and stakeholders of care; and person-centred care through a decolonial lens. To uproot inequity and create fairer and more respectful maternity care for women, birthing people and staff, it is vital that contemporary maternity institutions understand the decolonial perspective. This novel enquiry offers a scaffolding to undertake this process. Due to significant differences in colonial history between Western colonising powers, it is important to decolonise with respect to these different territories, histories and challenges.

## Introduction

Respectful maternity care (RMC) has developed organically through the last decade, with many global organisations concerned about human rights in maternity care, such as the White Ribbon Alliance, World Health Organisation (WHO), the International Federation of Gynecologists and Obstetricians (FIGO) and the International MotherBaby Childbirth Organisation (IMBCO), lending their efforts to develop various frameworks (Asefa 2021; Davis-Floyd et al. 2011; Lokugamage and Pathberiya 2017). The International Childbirth Initiative (ICI) which has been ratified by several global institutions now describes 12 steps to RMC (Lalonde et al. 2019). The ICI is concerned with maternal safety which is grounded in human rights and kindness and compassion as well as supportive working environments for staff (The WHO Reproductive Health Library 2018). However, it is also important to contextualise the concept of RMC as a reflection of societal attitudes, as in Dahlen et al’s 2022 Lancet commentary (Dahlen et al. 2022), where they say.

“The way we treat women during pregnancy, childbirth, and postpartum, and the institutional options of care we provide them within health systems, directly reflect the way we value women in our societies. In too many settings we are ignoring the benefits of midwifery models of care, degrading the status of midwives, and removing financing from midwifery services and education, under the guise of safety that ignores physiology and women's chances for optimal mental and physical health.”

Nevertheless, in order to identify the structural injustices in maternity systems, it is crucial to adopt a decolonial angle—to evaluate healthcare systems and structures through the eye of history in order to understand how these injustices have become so deeply ingrained over time. In this paper, we attempt to examine the barriers to RMC in the UK healthcare system through a decolonial lens. This involves recognising areas of inequity or tension within maternity care in the UK, derived from a Euro-American model of healthcare, which has deep roots in colonial medicine and has been extensively shaped by colonial-era hierarchies of power. As there are significant differences in the colonial histories of various European colonial powers across different geographic regions, and populations, it is important to decolonise with respect to these unique histories, territories and challenges, though there are areas of commonality and shared experience to draw from.

## Methods

### Data sources

The initial objectives of our literature review were to search for information in peer-reviewed journals about decolonising maternity care in the UK. This yielded very little. Thereafter, we drew from wider information bases (Table 1). Many of the knowledge sources were grey literature derived from inter stakeholder public engagement discourses. Furthermore, we drew from discussions with academic and lay commentators on human rights in childbirth. From this process, we observed the most active/outspoken lay signals:

1. Sanctioned ignorance (Wong et al. 2021) around the historic unconsented experimentation and treatment of black women and slaves in knowledge creation.
2. Intersectional maternal mortality rates where black and brown women are more likely to die during pregnancy and childbirth.
3. Cultural appropriation of indigenous therapies.
4. LGBTQ + , feminism and additive language in shaping a more inclusive maternity care space.

**Table 1:** Information sources used for article

PUBMED Search 1966 – 20 June 2021 Keywords used: Decolonising AND decolonial maternity, decolonising AND decolonial maternity UK, decolonising AND women’s health, decolonising AND obstetrics OR gynaecology
CINALH Search 1982 – 20 June 2021 Keywords used: Decolonising AND decolonial maternity, decolonising AND decolonial maternity UK, decolonising AND women’s health, decolonising AND obstetrics OR gynaecology
Written outputs from ‘decolonisation’ public engagement events in the UK
Discussions with expert research academics for reviews of the literature
Multimedia knowledge resources and grey literature from social media maternity posts and support groups
PhD theses
Personal experience participating in and writing several reviews of the decolonising healthcare literature

### Analytical method

The subject matter has arisen in an organic, non-linear accrual of knowledge. The approach of drawing information from disparate sources aligns with Dr Ijeoma Nnodim Opara’s PLOS blog ‘It’s Time to Decolonize the Decolonization Movement’ (Opara 2021) and has been used by one of the authors in a previous paper, *‘Decolonising ideas of healing in medical education’* (A. U. Lokugamage et al. 2020b). The authors suggest that the less formal method by which this knowledge has been gathered reflects the person-centred agenda of RMC. Women, alongside healthcare staff and students have led the movement towards RMC, sharing their concerns regarding oppression, injustice or bias from first-hand experience of the system, and presenting alternative solutions. The subjective and complex nature of these experiences make it difficult, if not impossible, to apply a hierarchical taxonomy towards evidence regarding the issues surrounding decolonising maternity care. Indeed, it prompts us to question the processes by which medical knowledge is produced, evaluated and canonised, and the types of knowledge and knowing that are excluded by these processes.

At the moment, there is a global debate about the nomenclature of minority groups where group terms risk homogenising very heterogeneous groups. In this paper, we have chosen to refer to minority ethnic groups instead of the term ‘BAME’ and used the term women/female when discussing the context of historic androcentric bias. We debate heteronormative bias using the terms woman and birthing person in the discussion that follows on Intersectionality. We do not intend to address the nomenclature any further in this paper as it would require robust excavation, exploration and scholarly inquiry into an emerging field that would warrant a separate article.

‘*Dismantling the Master’s House’*(Lorde 2007) is a symbolic term described by Audre Lorde, in reference to the process of disrupting the gradient of power imbalances within historical institutions, by seeking ‘tools’ from outside the orthodoxy of these institutions in order to undo the colonial legacies embedded within. This terminology was later adopted in a campaign by University College London (UCL),^1^ with the purpose of undoing persistent social and scientific inequalities seeded in a colonial past. These hierarchies perpetuate power imbalances that stem from colonial legacy, in which particular norms are upheld. As a consequence of the discrimination it exerted against disempowered and marginalised groups within colonised populations, colonialism left behind in its wake a legacy that was racist, sexist, heteronormative, classist and ableist. In this paper, we pay special attention to the relationship between coloniality, patriarchy and androcentrism in producing educational and societal norms, with reflection into enculturation into these norms. We describe how white male power has been centred throughout maternity care, to the detriment of women and their bodies. We also discuss the importance of prioritising public, staff and service user involvement in this debate, as decolonisation has historically been a bottom-up process, spearheaded by populations disadvantaged by power imbalances wrought by coloniality, rather than a top-down institutional policy imposed by those already in power.

The goal of decolonisation is not to merely mitigate the harms of coloniality but to fundamentally restructure the systems that continue to produce and perpetuate colonial power. Tuck and Wayne Yang (2012**)** prescient and powerful statement that decolonisation is ‘not a metaphor’ calls for activists in this arena to reckon with the radical implications of the decolonial framework. This entails going beyond performative initiatives by institutions, such as the establishment of equality, diversity and inclusion (EDI) agendas without sincere commitment of funding and resources to grassroots movements that can bring about lasting structural change. While words like *diversifying* have often been used tokenistically in such rebranding projects, *decolonising* is an unsettling process, an upheaval of an order established and deeply entrenched by coloniality in society today. Therefore, it is no surprise that the decolonial perspective is well documented as being discomforting (Matias 2016; Leonardo and Porter 2010; Matias and Mackey 2016) to those that have historically held power and/ or are unaware of the impact of colonial history on power hierarchies within healthcare. This discomfort has often been portrayed as a type of ‘fragility’ – that is, a state of insecurity that leads to a reactionary and often exaggerated defensiveness towards what is perceived as a threat to one’s identity and/or group. This has been expressed as *‘white fragility’*(Diangelo 2011), and in the context of this paper could even be called ‘*white coat fragility’* (A. U. Lokugamage et al. 2020b). More recently, Skopec and colleagues offered the term ‘*epistemic fragility*’(Skopec et al. 2021) to describe the resistance of higher education institutions to perspectives that challenge the status quo and go beyond established epistemes. This notion is particularly relevant within medicine in the UK, in which the biomedical perspective is not only privileged, but predominant – resulting in a system ruled by a ‘biomedical hegemony’(Baer et al. 2003), rather than characterised by medical pluralism.

In this paper, while acknowledging that discussions cannot be divided into discrete areas of inquiry, we nevertheless offer a scaffold to form an epistemology for examining the issues and provide the reader a basis from which to begin considering what it means to decolonise maternity care and deconstruct their own assumptions about what maternity care ‘should’ be. We have delineated three broad areas of inequity or tension within maternity care in the UK and their roots in Britain’s colonial past and present:

I. Professional structures and institutional biases.
II. Power imbalances between types of staff and stakeholders of care.
III. Person-centred care through a decolonial lens

## Results

### Professional structures and institutional biases

#### The androcentric bias in obstetrics and gynaecology

What we term as ‘Western’ medicine, grounded in centres of influence throughout North America and Western Europe, was established in a period of history characterised by patriarchy and male dominance – the legacy of which is experienced in the present through *systemic bias* and *unconscious bias*. Androcentrism is a term used by many midwifery scholars to describe the male dominance of Western science and medicine. Historically, it was adopted by midwives to describe the culture in maternity care, and it evolved as what was deemed a less provocative descriptor of patriarchy (D. J. Walsh 2010a; Denis Walsh and Steen 2007; Deery 2003; JERVIS BK 2019). The history of obstetrics has been narrated through an androcentric and male-oriented discourse, where women were seen as helpless (“the weaker sex”) and their bodies – including its natural processes—pathologised, thereby warranting medical surveillance and intervention. Reflecting this sentiment, Professor Ian Craft said in the 1980’s that “*Childbirth is as much a natural event as is death, but whether it should be allowed to be so is a different matter*” (Murphy-Lawless 1998). In response to this attitude by obstetricians, a ‘*Birthrights Rally’* of 5000 protesters was held in 1982 around the Royal Free Hospital, Hampstead, London, UK, organised by Janet Balaskas (*Active Birth* founder) in collaboration with Sheila Kitzinger (birth anthropologist), and childbirth organisations such as AIMS (Association for Improvements in Maternity Services), NCT (National Childbirth Trust) and Radical Midwives. At the protest, Sheila Kitzinger stated how “*the incident in the Royal Free* [Hospital] *was a goad to political action to challenge the ‘male dominated model of childbirth, of the need to topple the autocratic control of a profession directed towards the treatment of pathological conditions’*” (Murphy-Lawless 1998).

Today, although 55% of the RCOG workforce is female (RCOG 2018), one still needs to be mindful of the aforementioned knowledge bias in sciences, arts and humanities publications that can be transmitted through the *hidden curriculum* in medical training. The *hidden curriculum* is defined as “*the unwritten, unofficial, and often unintended lessons, values, and perspectives that students learn in school*” (The Glossary of Education Reform 2015). While the *formal curriculum* consists of the courses, lessons and learning activities students participate in, as well as the knowledge and skills educators intentionally teach to students, the *hidden curriculum* consists of the unspoken or implicit academic, social and cultural messages that are communicated to trainees while they are in education (Lempp and Seale 2004). It is conceivable that despite being a female doctor, through the insidious process of the *hidden curriculum,* it may result in unconscious androcentric bias in the conceptual framework of medicine and women’s health (Cahill 2001). As this bias is so deeply embedded in training and working environments, it escapes attention and remains preserved by institutions that are resistant, even impervious to external change. Indeed, this is witnessed in the legal case of *Montgomery v Lanarkshire* [2015] where Lady Hale referred to the historical medical paternalism in obstetrics, in a case regarding a patient wanting a medical intervention but denied it by a female obstetrician.^2^ The word ‘patient’ in this context is used as in the legal report. The sociological discourse about the tension between the androcentric, biomedical, technical model of childbirth, versus the feminist, social and embodied model of birth is rarely represented in maternity, biomedical journals or textbooks. In Walsh’s paper ‘*Childbirth embodiment: problematic aspects of current understandings’* he articulates that “*the omission [of this discourse/tension] has had negative consequences for consumers and professionals alike. In particular, the arena of childbirth has been colonised by contrasting approaches to the body that stifle the realisation of humane maternity care*” (Walsh 2010). In such a setting, the RCOG Women’s Network could prove to be an important decolonial influence in the advocacy of woman-centred perspectives and person-centred pathways through the historical binaries of medicalised versus natural childbirth. We discuss this area later with respect to stakeholder empowerment.

#### The colonial legacy within higher education institutions

More higher educational establishments have been engaged in reviewing their links to the slave trade (Glasgow University),^3^ eugenics (University College London)^4^ and the colonial oppression in global research (School of Oriental and African Studies, SOAS).^5^ Prompted by ground-up, member-led movements, some institutions have reflected on their relationship to benefactors with links to slavery and colonialism, some of who have been commemorated by statues and designated spaces within the grounds of these institutions. The Royal Colleges of the various medical and surgical specialities within the UK, including the Royal College of Obstetricians and Gynaecologists (RCOG), have yet to lead in decolonial evaluations of their institutional histories. Without reflection on the colonial history regarding the acquisition of knowledge throughout the evolution of modern medicine within undergraduate and postgraduate syllabi (Turner et al. 2014; Chow, Lokugamage, and Gishen 2019) institutions run the risk of unintentionally perpetuating persistent academic inequities and tacit acceptance of medical knowledge based on eugenics or colonial exploitation. This has parallels to the call for schools in the UK to teach students a broader perspective of colonial history, to provide a more honest account of the injustices committed under the banner of British imperialism.^6^

One specific area of knowledge in maternal care that has been acquired through colonial oppression of black slaves has been highlighted in the press (White 2020). Urogynaecology techniques to repair vesico-vaginal fistulae (a complication after obstructed labour in childbirth) and the Sims speculum were developed in America during the time of colonial enslavement of black people. An obstetrician and gynaecologist, J. Marion Sims (Spettel and White 2011) developed the instrument and surgical techniques through experimentation on black women slaves, without their consent and without using anaesthesia. This knowledge was historically and tacitly absorbed into the knowledge base that underpins the obstetric and gynaecological paradigm without question. However, decolonial activists have called on the RCOG to demonstrate a decolonial stance on this knowledge by honouring the black slaves in some way (Downes 2020). Activists feel that honouring the black slave women would signal a strong anti-racist message from the RCOG, in the face of structural racism as discussed in the BMJ 2020 special edition on Racism in Medicine (Kmietowicz 2020). Sim’s technical knowledge has until now been transmitted to trainees without any reference to the unethical and violent methods by which it was obtained. There have also been recent controversies around the standardisation of urogynaecology ‘mesh’^7^ insertion without adequate research studies to prove its efficacy and safety. This was followed by an Independent Medicines and Medical Devices Safety Review (IMMDS Review) chaired by Baroness Cumberlege in 2020 called ‘First Do No Harm’(Cumberlege 2020) that directly draws from the lived experiences of people harmed that led the authors to conclude that the health care systems are.

*“disjointed, siloed, unresponsive and defensive. It does not adequately recognise that patients are its raison d’etre. It has failed to listen to their concerns and when, belatedly, it has decided to act it has too often moved glacially. Indeed, over these two years we have found ourselves in the position of recommending, encouraging and urging the system to take action that should have been taken long ago.”*

This report is enacting a decolonisation process of disrupting ingrained power hierarchies. In a BMJ summary article,

“What the Cumberlege team has flagged is the stubborn flaw that lies at the heart of the practice of medicine. It is often called “culture.” But this type of embedded attitude seems to go beyond culture, beyond fear of liability, and beyond the profit motive when that exists. It is a patronising and insufficiently curious way of doing business that is often at odds with the realities of helping patients heal and is increasingly out of place in a connected modern world.”(Haskell 2020).

From a medical ethics, perspective obstetricians have much to benefit from reflecting on the story of Sims and the IMMDS Review of Cumberlege, especially in developing new surgical techniques.

#### Decolonising the medical and midwifery training

##### Undergraduate teaching

Maternity care cannot be separated from the life cycle of women and the medical conditions that can affect them during pregnancy, so the discriminatory biases described in other areas of medicine and even gynaecology are relevant to mortality and morbidity in maternity care. Student–staff groups across various medical schools across the UK, including UCL Medical School through its decolonial work,^8^ have signalled a dearth of teaching material that represents how clinical signs present in ethnically diverse people with varying skin tones, which of course, includes maternity care. Medical schools do not routinely teach how cyanosis looks like in black people in medical training and the accuracy of pulse oximeters are affected by skin pigmentation (Hidalgo et al. 2021). Breast conditions, like mastitis, have different appearances in different skin tones (Boutet 2012). Black women are less likely to be diagnosed with endometriosis compared to white women (Bougie et al. 2019), and ethnic minorities are more likely to die from skin conditions such as melanoma than their white counterparts (Cormier et al. 2006). While there is an extensive body of literature detailing the ethnic variation of reference ranges for biochemical and haematological markers (Tahmasebi et al. 2018), Cerdeña et al. say in their Lancet article.

“Emerging scholarship underscores the harms of these race-adjusted practices, even as some continue to defend them, touting their ability to capture yet-understood differences in clinical measures between racial groups. However, propagation of race-based medicine promotes racial stereotyping, diminishes the need for research identifying more precise biomarkers underpinning disparities, and condones false notions about the biological inferiority of Black and Brown people. Hence, even if significant findings or clinical anecdotes support the use of racially tailored practices, they should be rigorously critiqued and mediating variables, such as structural conditions, should be analysed accordingly.”(Cerdeña et al. 2020)

The above arguments were used in the paper ‘*Racial profiling for induction of labour: improving safety or perpetuating racism*?’ to challenge potential assumptions in the NICE guidelines proposed draft (2021) on induction of labour which disregarded the existence of structural racism as a determinant of health in maternity care (Douglass and Lokugamage 2021). Some ethnic variation might be accounted for by epigenetic variation that arise from differences in social environments and upbringing. One example of this is the phenomenon of ‘weathering’ (Sullivan 2013; Simons et al. 2016), a hypothesis that poorer health outcomes in minoritised ethnic groups may arise in part from epigenetic changes that occur at a cellular level, due to chronic exposure to the effects of socioeconomic positioning, prolonged hardships and discriminations in society. Furthermore, there needs to be awareness that minority ethnic groups are under-represented in clinical trials (Hussain-Gambles et al. 2004) and the resulting bias in data does not accurately reflect the whole population. Lack of representation of these arguments in pedagogical material can create diagnostic challenges insofar as medical conditions in minority ethnic groups are concerned. Whenever there are systematic delays in diagnosis or misdiagnosis occurs, patient safety is at risk as poor prognoses, and long-term health outcomes can be expected to follow. The *Mind the Gap* handbook (Rimmer 2020) of clinical signs in black and brown skin, is a recent decolonising initiative that can help clinicians to identify conditions in darker skin tones. The British Association of Dermatologists are actively improving their educational tools in this area (British Association of Dermatologists. 2022a, 2022b). More resources like these must be developed to create a more inclusive medical curriculum across all specialties that better reflects the diverse patient population we serve.

##### Postgraduate teaching

The nuances of decolonial education described for undergraduate teaching needs to be embedded in postgraduate and professional development programs. There also continues to be an ethnic *attainment gap* in professional training education. This is well documented in both undergraduate medical training and postgraduate RCOG training (Woolf et al. 2016; GMC 2018; Joint Royal College of Physicians Training Board 2018; Shah and Ahluwalia 2019; Woolf et al. 2011) where there is a differential in awards and attainment and progression for minority ethnic students. In the *British Medical Journal* (BMJ) edition 2020 on Racism in Medicine, Woolf explains that this is thought to be of a perceived lack of inclusion in extra educational opportunities within educational organisations for minority ethnic students and trainees (Woolf 2020). More work is being done to unpick this phenomenon and find appropriate solutions (RCOG 2022). Racism in the context of postgraduate training in obstetrics and gynaecology is part of the decolonising agenda—particularly as many graduate doctors may originate from ex-colonial countries and are experiencing discrimination (WRES Implementation team 2021). The Ockenden Report 2022 does give a decolonial signal of the continuing struggles and inequities of these type of doctors (SAS doctors) in the maternity care system. These inequities include support in their jobs, attainment, career progression, rota hours and time for learning opportunities. So greater structural equality is essential(Department of Health and Social Care 2022) for more obstetricians from minority ethnic backgrounds to be empowered to join the discourse and help shift cultural attitudes at the grassroots level by advocating for culturally safe measures (Douglass and Lokugamage 2021) to improve maternity outcomes.

We have been unable to find data about any investigation into whether an *attainment gap* exists in midwifery, and this is an area that needs further work from midwifery higher educational establishments. However, there are encouraging decolonising projects afoot such as the October 2020 edition of the *Student Midwife Journal*^9^ where each article was written by black or brown authors tackling topics such as racism, Black Lives Matter, and cultural sensitivity.

##### Epistemic and geographical bias in knowledge production

In addition to the biases present in institutions and medical/midwifery curricula, there is also clear evidence that scientific literature is dominated by publications and research outputs from the Global North^10^(Pan et al. 2012). Thus, knowledge is incorporated into practice from those centres, which historically have had more white male Eurocentric influences, to the exclusion of others. Hence, the Global North exerts more power and influence than the Global South in research generation, which skews what we think is scientific fact or opinion. This geographic bias is well documented in decolonial scholarly output (Skopec et al. 2020; 2021; A. U. Lokugamage et al. 2020b).

With the Global South being largely under-represented in research, our understanding of childbirth is skewed, and the lived experience of non-Eurocentric or indigenous women that influences childbirth is marginalised or excluded from the maternity care discourse. The majority of research on childbirth is on pathology (Walsh 2010), and it, therefore, only gives a reductionist view of the female body, which is viewed akin to a perpetually faulty machine that requires continuous maintenance and intervention in order to function smoothly (Hamberg 2008; Holdcroft 2007). It has been established that there is a gender bias in medicine. In Gabrielle Jackson’s book Pain and Prejudice, she comments on research in this field by saying “*For much of documented history, women have been excluded from medical and scientific knowledge production, so essentially we’ve ended up with a healthcare system, among other things in society, that has been made by men for men*” (Jackson 2019). This medicalised bias justifies obstetric intervention for successful childbirth. However, from an evolutionary standpoint, we know that natural childbirth is a reliable biological process, with technological births reserved for complicated deliveries. An overwhelming focus on reproductive pathologies and medical technologies misses the unique and varied experiences of childbirth upon which relationally focused, humanised treatments are created.

When reflecting on the obstetric cultural impetus to mechanically tinker with the natural physiological processes of childbirth, the ARRIVE (A Randomized Trial of Induction Versus Expectant Management) trial, gave the obstetric community justification to ‘normalise’ labour induction at 39 weeks, in order to reduce the rate of Caesarean deliveries (Grobman et al. 2018)(Davis et al. 2020); to note, this trial measured short-term outcomes. However, more recently, a 16-year follow-up midwifery research study has been published which examines the consequences of induction of labour and showed long-term adverse sequelae. Induction of labour:

“for non-medical reasons was associated with higher birth interventions, particularly in primiparous women, and more adverse maternal, neonatal and child outcomes for most variables assessed. The size of effect varied by parity and gestational age, making these important considerations when informing women about the risks and benefits of IOL”.(Dahlen et al. 2021)

Again, when considering intrusive new interventions, the OASI (Obstetric Anal Sphincter Injury) Care bundle was introduced at many maternity services after studies showed that it can reduce rates of OASIs from 3.3% to a 3% (Gurol-Urganci et al. 2020). One aspect of the care bundle involves a per rectum examination to assess the perineum, even when it appears to be intact. Midwives and obstetricians alike have raised their concerns, questioning whether the increase in indignity/*obstetric violence* (Thornton and Dahlen 2020; A. U. Lokugamage and Pathberiya 2017) is worth such a small gain. While there is a body of literature that highlights the role of more holistic approaches in reducing OASI’s^11^ and wider evidence that highlights the effectiveness of midwifery-led continuity models of care in improving maternity outcomes (Sandall et al. 2016), an interventionist approach continues to prevail in maternity care, exemplifying a blindness to explore these individualised, person-centric and midwifery-led care models outside the realm of obstetrics.

From a global perspective, there is now an international discussion about re-centring displaced indigenous midwifery or doulas in a desire to humanise birth. A documentary on the resurgence of Australian aboriginal midwifery vividly portrays this and also the focalising of birth in terms of planetary sustainability/ecology. The documentary is called ‘Birthing On Country. DJÄKAMIRR: Caretaker of Pregnancy and Birth’.^12^ In Sarah Sunshine Manning’s article on decolonising birth in a Native American setting, she quotes a Navajo midwife saying “*A lot of the time in hospitals, people don’t approach women in a way that says to them that they are the centre of the birth, or in a way that gives the woman control*” (Manning 2018). To not just include but to platform the voices of the Global South, there is a need for *cultural humility* in planning global health projects from UK global health funding organisations, such as the RCOG, Wellcome Trust etc. One must be mindful of historic power imbalances in the research agenda of women’s health, whether it is related to race, sexism, classism or other historical inequalities, all of which were oppressed in colonial rule or pre-existing biases within the developing country (Kymlicka 2018) which may still dictate and adversely affect research agendas and knowledge production. It is prudent in global research collaborations between the Global North and South, to make sure that there is local expert and public involvement at all stages of the research and also have ethics committees from the Global South, to offset historically derived and structurally enabled the power imbalances. Creative space should be given for ideas or hypotheses for projects from grassroots needs, rather than neocolonial agendas where benefits of the project can be more heavily weighted towards the Global North (Fayemi and Adeyelure 2016; Cordeiro-Rodrigues 2020; Chimakonam and du Toit 2018). It is important, however, to be mindful of endemic issues in some Global South countries where women’s voices continue to be marginalised.

### Power imbalances between types of staff and stakeholders of care

#### Decolonising midwifery

Midwifery is a philosophy, far more ancient than the obstetric profession, which has been practised around the world and supported women through childbirth for centuries (Ehrenreich and English 2010). Similarly, in much of the Global South, female midwives have long been the sole caregivers for pregnant women and women in labour. Here in the UK, the lived experiences of the ‘*Windrush’* midwives, who have been an important part of the maternity care NHS workforce, were explored by Daniel Olusoga in the BBC series *Black and British: A Forgotten History*.^13^ It would be a worthwhile project to actively record their lived experiences as black midwives, as there seems to be a paucity of academic papers detailing this history.

Within the colonial era, midwifery became increasingly ‘colonised’ by obstetrics, leading to a paradigm shift towards medicalised delivery techniques and as a consequence, worldwide adoption of the lithotomy birthing position for obstetric convenience (Dundes 1987). Jo Murphy Lawless (sociologist) in her book *Reading Birth and Death* states that

“Stemming from this singular focus, their descriptions of pregnancy and birth and the definitions of how the female body worked, limited the scope of who might intervene to help a woman and how. They became the sole experts and, in their pursuit of the power accruing to experts, they routed all other birth attendants: family members, traditional handywomen and traditional midwives”.(Murphy-Lawless 1998)

In the eighteenth century European age of enlightenment, as obstetrics became more professionalised, midwifery was further delegitimised and their practices demonised as witchcraft, with many of them sent to their deaths (Ehrenreich and English 2010). The eventual professionalisation of midwives and the advent of academic midwifery, as well as, subsequent evidence-based publications about midwifery care models, have supplied undeniable clinical and sociocultural backing for the overarching benefits of midwifery care (Vermeulen et al. 2019). However, one could question whether the process of professionalism is in itself a legacy of colonialism. When a role based on experience, such as midwifery, which is natural, flowing and resists classification or hierarchical order, is formalised or ‘shoe-horned ‘ into a structure, the very attributes which makes it effective are stripped away. Thus, the historic role played by midwives passed through time and with their own cultural particularities can be stifled.

Labour and birth services are structured around a predominantly obstetric model (Machin and Scamell 1997). This has led to a historic power imbalance between obstetricians and midwives with obstetricians holding more power. There is a history of oppression of midwifery at various timepoints and geographical locations, in the UK and US, as well as, Germany, France and Spain (Marland 1994). In the UK, although midwifery has endured eventually as a parallel speciality, the residual power imbalance between obstetrics and midwifery lingers. This can be exerted as an oppressive constraint on midwives who want to practice in a supportive ecological style and are trying to minimise nonessential intervention in physiological birth in the face of the increasingly medicalised practice of maternity care.

In some ways, this power struggle between obstetrics and midwifery can be regarded as a struggle between embodiment and essentialism;(D. J. Walsh 2010b) midwives being the experts in the ecology of birth and obstetricians the experts in the technology of birth. Both are important in achieving safe and effective maternity care provided that there is mutual respect of complementary roles and person-centred autonomy in decision making is maintained.

However, we need, at this point, to reflect back to the legal case of *Montgomery v Lanarkshire* where there was a human rights violation by under medicalising care through not actively listening to the patient and collaboratively creating a treatment plan. Enabling both medical and ecological frameworks of birth within a person-centred, respectful, compassionate environment is essential to achieve safe and respectful maternity care. So when we examine recent health care crises with tragic outcomes to patients in the UK, such as highlighted in the Kirkup (Kirkup 2015) and Ockenden Reports (Department of Reproductive Health and Research, World Health Organization, 2017), what would be optimal is that in addition to local issues/failings, this analysis is set against national population statistics, systematic reviews of models of maternity care, short- and long-term implications, as well as a decolonial lens. The decolonial lens should be applied at both the structural and interpersonal level which would give a depth of analysis to workplace conflict, lack of multidisciplinary team working and mutual respect across ingrained power hierarchies impacting on staff, patients and service provision.

We should also acknowledge that when we over generalise about obstetricians and midwives, we lose the understanding that within each profession, there are a spectrum of views, beliefs, practice and ways of working regarding the conceptual extremes of a mechanistic model of birth versus an ecological model. The decision to relocate the Royal College of Midwives (RCM) into the same building as the RCOG^14^ could be a double-edged sword. On one hand, it could be seen to increase collaboration and help flatten power imbalances, provided that there is sharing of ideas and information through joint collaboration and equal partnership to achieve high-quality, person-centric care. We have seen that this sharing of physical space has led to a publication that provided guidance on the provision of physiological births at home and midwifery-led centres during the COVID-19 pandemic (Royal College of Midwives and Royal College of Obstetricians and Gynaecologists 2020). However, on the other hand, great care needs to be taken in this re-location so that historic power imbalances are not augmented.

#### Redistributing power to stakeholders and re-centring their voices

The appointment of the first female president of the RCOG in 64 years between 2016 and 2019, Lesley Regan, led to profound positive changes in the redistribution of power for maternity stakeholders. This could be seen to herald the start of an erosion of the androcentric historic biases previously discussed. This was demonstrated by allowing a women’s voices conference (2017) organised by passionate service users to be held at the RCOG. At that conference, officers of the RCOG sat through difficult testimonies of service users actively listening to critiques of the College and responded with a resolve to make improvements. The advent of the Women’s Network and Women’s Voices Involvement Panel within the RCOG, and the rise of the Maternity Voice Partnership (MVP),^15^ bolstered by general NHS agenda outlined by the patient experience framework, also occurred during this timeframe (NHS National Quality Board 2012).

The Maternity Voices Partnership (MVP) by NHS England is an NHS working group where a team of women and their families, commissioners and providers (midwives and doctors) work together to review and contribute to the development of local maternity care. They are supported by a group called National Maternity Voices (NMV) where resources have been allocated to facilitate the voices of services users to be heard and shape their local service. There is an intervention called ‘walk the patch’ where a local MVP (Maternity Voices Partnership) member is allowed 1 h to walk through their maternity unit and ask for feedback from women. It is possible for them to witness care pathways at work, for instance how women are admitted for caesareans, taken to theatre and leave theatre. This is so they can give feedback about how the user experience is truthfully experienced and can work on co-produced solutions that can only be seen from a grass roots perspective. The evolution of the MVP has been a positive step in flattening power hierarchies in maternity service, which many other countries may not have heard of. It demonstrates that, even though it is not badged as decolonisation, such structural reflexivity and active willingness to empower maternity users to co-produce services, is possible.

### Person-centred care through a decolonial lens

#### Women’s voices, autonomy and power

Listening to the many and diverse voices of women who use maternity systems is a core pillar of decolonisation. It symbolises the ground-up influences required to vocalise what top-down equality and diversity institutional initiatives may miss or unintentionally omit. The importance of patient experience has become an essential component of the National Health Service (NHS) especially after the health care scandal that led to the *Francis Report* in 2013 (Francis 2013; NHS Improvement 2018). As mentioned, the RCOG has developed its Women’s Network and Women’s Voices Involvement Panel which has the power to influence policies, guidelines, conferences and postgraduate education.^16^ All of these initiatives are decolonising stepping stones but the challenge will be to hear the voices of marginalised maternity service users. In the wake of the Supreme Court ruling in the obstetric case of *Montgomery v Lanarkshire*, patient autonomy (the right to accept or refuse medical interventions) and by that healthcare human rights in general is strengthened through case UK law. This legal case could be interpreted as a decolonising force. In the case Lady Hale said “*social and legal developments which we have mentioned point away from a model of the relationship between the doctor and the patient based upon medical paternalism*”. This refers to the lingering but unconscious historical patriarchal norms in obstetric practice and in medicine in general.

The case against medical paternalism may be further strengthened through a critical evaluation of the clinical recommendations framed as ‘evidence-based medicine’, which are often routinely applied in practice. Analysis of specialist guidelines published after 2007 by the RCOG by Prusova and colleagues in 2014 (Prusova et al. 2014) found that only 21 per cent of these were backed up by the high-quality evidence (Grade A and B), based on results from systematic reviews or randomised control trials. The remaining majority of these guidelines were based on weaker evidence from case control studies, case reports, ‘bench research’, i.e. basic science research before clinical application and the largest proportion is from expert opinion only. Similar findings have emerged in analyses of equivalent guidelines in America (J. D. Wright et al. 2011), Canada(Ghui et al. 2016) and across other specialties(J. M. Wright 2007). As highlighted by *Montgomery v Lanarkshire*, doctors have a legal duty to ensure that patients are fully aware of the risks of each treatment option and given the choice of alternatives. Therefore, transparency around knowledge production and areas of uncertainty in the development of clinical guidelines is essential to ensure informed consent may be obtained at every step of care. This is essential to enabling shared clinical decision making in a way that prevents abuses of power by healthcare institutions and professionals and preserves women’s autonomy.

#### Intersectionality in maternity care

*Intersectionality* describes how various axes of discrimination on the basis of race, class, sex, gender and disability converge to produce multiple and compounding levels of oppression. This term was first coined by Professor Kimberlé Crenshaw to understand the multiple oppressive forces faced by African American women in everyday life (Columbia Law School 2017). Maternal mortality and racial inequity in death rates are considered decolonisation pressure points. This can be identified in the *2018—2020 Mother and Babies: Reducing Risk through Audits and Confidential Enquiries* across the UK (MBRRACE-UK) reports (Knight et al. 2018; Knight et al. 2020; Draper et al. 2021), and similar reports in the USA (Angley et al. 2016), as well as the UK Obstetric Surveillance System (UKOSS) study on COVID-19 deaths.^17^ Black feminists have been strongly voicing their experiences of structural racism and intersectionality (Nash 2008), as their symptoms and concerns have been brushed aside and trivialised.

This disturbing finding has been brought to the attention of the RCOG. As a result, there has been active action through their International Women’s Day event 2020 *‘We need to talk about race’,*^18^ as part of a collective response to the statistic that *black women are five times more likely to die in childbirth than white women, with mixed race women three times more likely and Asian women twice as likely to die compared to white women*, revealed in the confidential enquiry into maternal deaths (Knight et al. 2018). Following on from this event the RCOG has established a taskforce which has started collaborating with the *Five X More* anti-racist patient activism group to focus on reducing the adverse health outcomes experienced by minority ethnic women in the UK.^19^ Collaborative working with FIVEXMORE has produced ‘The Black Maternity Experiences Report’. This Report as well as a separately published report by maternity human right charity Birthrights called ‘Systemic Racism, Not Broken Bodies’ now provide solid evidence of structural racism (Birthrights 2022; Peter and Wheeler 2022) in 2022.

A *Cultural Safety* model of care adapted to a UK healthcare system has been put forward as a way of reducing *unconscious bias* and racism in maternity care Lokugamage and Meredith 2020; Lokugamage et al. 2021b). The specifics of which are laid out in the Cultural Safety tree infographic (Image 1) within the paper ‘Translating Cultural Safety to the UK’ which may be useful for organisations beyond maternity care to utilise. The concept of *Cultural Safety* has its roots in New Zealand Maori nursing education (Papps and Ramsden 1996) and aims to undo the systemic biases in healthcare by acknowledging the inherent power imbalances present in ‘health care user’ vs. ‘provider’ relationship. Such an approach would require willingness and active participation on the part of doctors and midwives to critically reflect on their inherent preconceptions of ideal care and to acknowledge their privileges, biases and the power imbalances present in maternity care (Laverty et al. 2017).

**Fig. 1:**
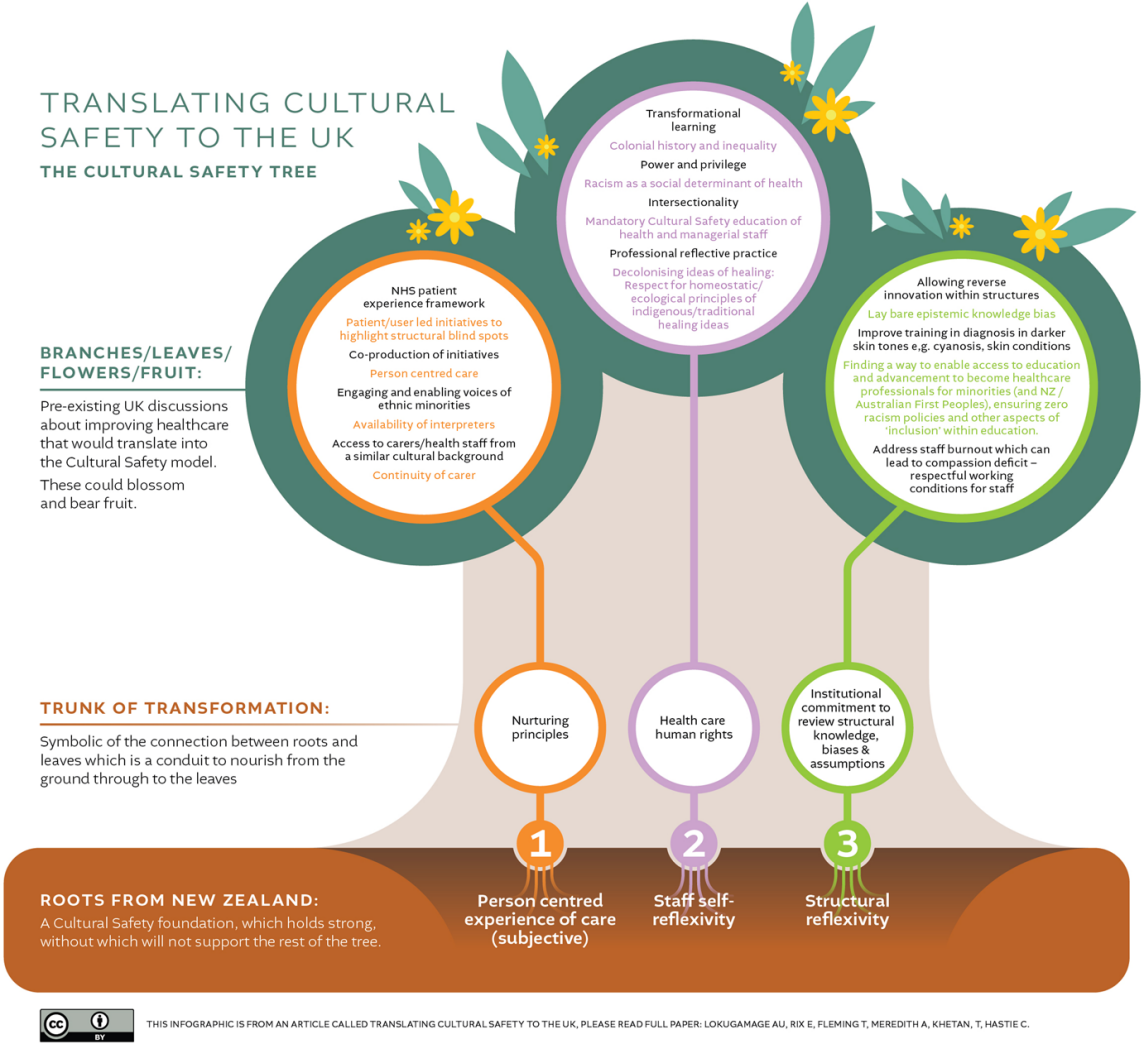
Cultural Safety Tree

At the time of this article’s submission, we note the global debate around the proposed introduction of the Māori term 'whānau' by the Midwifery Council in Aotearoa/New Zealand to deliver family centric care^20^ which are designed to empower families as a whole.^21^ The deep interconnective sociocultural-spiritual construct of ‘whānau’ has no simplistic direct corollary in coloniser concepts. So, this is a very complex and developing area.^22^

The heteronormative legacy of colonialism has had a part to play in health inequalities and poorer experience for LGBTQ + people in our health and social care systems in the UK.^23^ For instance *“British colonies were much more likely to have criminalization of homosexual conduct laws than other colonies or other states in general. This result holds after controlling for other variables that might be expected to influence the likelihood of repressive lesbian, gay, bisexual and transgender (LGBT) rights legislation”*(Han and O’Mahoney 2014)^24^. Subtle homophobia has been identified by same sex parents engaging with maternity services (Hammond 2014) as well as the impediment in the involvement of the co-mothers and the invisibility of the lesbian mothers due to heteronormativity. There has also been a generational shift in attitudes within the LBGTQ + communities. Present-day younger generations have found diverse expressions with respect to gender and sex, than at any other time in history, preferring to adopt a more inclusive language (DeAngelis 2002), which could be replicated by the historical institutions, such as the health sector, if they are to serve the community effectively (NHS digital service manual 2020) (LGBT Foundation 2022). The LGBT Foundation for says:

“Trans men and non-binary people often report challenges in navigating maternity services, whether this is due to the highly gendered language used within these spaces, or to the common occurrences of personal misgendering by maternity service professionals. Additionally, there are also instances where maternity service professionals may not have had opportunities for training, and may not have even considered that trans men and non-binary people may want to become pregnant”(Petch 2020)

In social media around maternity, services conflict has arisen with respect to the terms of identification and address of persons, in finding an inclusive way to recognise *transgender, gender fluid or non-binary and other persons* in a maternity care setting, where historically the term *woman* has been used. This paper describes historical intersectional prejudices experienced by many groups that beset maternity care. Conflict between marginalised groups dilutes efforts to upend greater structural obstacles in healthcare. Steering towards a languages solution that does not oppress any particular marginalised group may benefit all parties. Additive language may be helpful in the process of navigating the endless circling of polarised arguments for and against unifying gender-neutral terms for all. Hence, language such as *women* and *birthing people; or breastfeeding and chest feeding* may be a suitable alternative—thereby respecting the cultural background of historically oppressed groups. However, what is important is for people to self-identify and that decision to be respected, with no oppression of either women or birthing people. Furthermore, it may be that narrative-based restorative justice processes around inclusive language might be a helpful and a compassionate approach to reaching agreement.(A. U. Lokugamage and Pathberiya 2017).

Consequently, we suggest a model of care that is individualised, embodying person-centred care, involving *Cultural Safety* where the unique identity of each receiver of care is respected. Care givers should consider their own biases and proceed in an individual-centric manner. Language can disempower service users; therefore, phraseology which reduces health inequalities and improves inclusion, is important and requires mindful speech. It is essential to find out the preferred manner in which each person wishes to be identified or their preferred pronoun. Ultimately, in addressing and dealing with any persons receiving care, practitioners should be trained to meet the changing sociocultural attitudes of the time and organisational systems should be optimised according to the diverse needs of each the woman or birthing person.

Terminology around gender and sexual identity and biological sex are areas that are evolving in the UK as well as globally (Gribble et al. 2022). It will be interesting to see how the discussion may be challenged to be more inclusive, and we anticipate that new developments will be made in the coming years to decolonise healthcare in both our social understanding and articulation of issues surrounding identity and biological sex issues, as well the different forms of self-identity and expression. As such, in this article, we use the term *woman/female* in discussion about androcentrism, and ‘person’ in the discussion about decolonising heteronormativity but acknowledge that heated discussions/debate are afoot in terminology in UK maternity services.^25^ In 2022, the Royal College of Obstetricians and Gynaecologists opened a consultation on a draft guideline relating to the care of transgender and gender diverse people (TGD).^26^

#### Humanising the mechanical model of the biological female body

The paternalistic epistemology of maternal care that arose from experimentation on colonised black women, has influenced the production of knowledge about how a biologically female body behaves during parturition. Examining the history of the partogram (a graphical model of the progress of a woman in labour and the dilation of the cervix over time) demonstrates this. The partogram is one of the corner stones of management of a woman in labour within maternity units in the UK. However, looking through the lens of decolonising women’s bodies, Lavender et al.’s *Cochrane Review*(Lavender et al. 2018) on ‘*Effect of partograph use on outcomes for women in spontaneous labour at term and their babies*’ spell out the colonial origins of this intervention and also the introduction and the persistence of this intervention without high-quality evidence. This *Cochrane Review* states

“The first obstetrician to describe the progress of labour graphically was Friedman,(FRIEDMAN 1954) following his study of the cervical dilatation of 100 African primigravidae at term. The women were given frequent rectal examinations and their progress was recorded in centimetres of dilatation per hour, producing a slope resembling a sigmoid curve ('S' shaped). This became known as the cervicograph. In an attempt to utilise midwives efficiently in a hospital and clinic service in Zimbabwe (then Rhodesia), where doctors were in short supply, Philpott 1972(Philpott 1972) developed a partograph from this original cervicograph. This provided a practical tool recording all intrapartum details, not just cervical dilatation. An 'alert line' was added following the results of a prospective study of 624 women.(Philpott and Castle 1972) The alert line was straight, not curved, and was a modification of the mean rate of cervical dilatation of the slowest 10% of primigravid women who were in the active phase of labour.”

Further modifications of the partogram *e.g.* Studd (Studd 1973) and WHO 2003(WHO 2015) etc. took place again without high-quality evidence. The findings were extrapolated to all women without any nuance regarding demographic characteristics, states of nutrition, location of birth or the quality of care without any randomised controlled trials. At this time, midwifery voices were not involved and power laid in the hands of obstetricians both in respect to income, status and academic standing.^27^ More recently, separate to the obstetric literature, midwives and doctors sympathetic to supporting physiological birth, have published observations of labour patterns in women cared for outside a hospital setting (birth centre or homebirth in high income countries) as being different and non-linear, indicating that normal physiological labour can progress or even go backwards depending on psychobiological effects of labour hormones 28(Albers et al. 1996; Albers 1999). These hormones are highly influenced by the women’s labouring environment and relationship with the care givers – similar to other mammals^29^(Groeschel and Glover 2001; D. Walsh 1994; Denis Walsh 2006; S. J. Buckley 2015; S. Buckley and Moberg K., n.d.; Gaskin 2003). Instead of seeing a low risk woman’s labour as a natural event which has its own rhythms and flows and which is allowed to unravel in its own way and time, by viewing the woman and childbirth through a mechanised lens, the woman is stripped of any inherent birthing competence and interfered with as if she were a machine for the perceived benefit of institutional efficiency. While mechanising may have its benefits in both simplifying a complex phenomenon and replicability, in so doing it loses the essential qualities valued by women. However, we acknowledge that RMC-based systems need to be in place to highlight escalation of risk status and access to appropriate interventions for women whose labour changes from low risk to high risk, to avoid harm. This is captured in the International Childbirth Initiative (Lalonde et al. 2019). The implications of not doing this has been laid bare by the Ockenden Report 2022 reviewing the failing of a UK Maternity care crisis in The Shrewsbury and Telford Hospital NHS Trust.(Department of Health and Social Care 2022).

The partogram’s mechanistic model of women’s bodies and the desire to process intrapartum care in an assembly line fashion in order to utilise institutional space, optimise staff, resources and standardise care is a contributory factor to *obstetric violence –* a term noted by the WHO(Department of Reproductive Health and Research 2017) and experts in human rights in childbirth.(A. U. Lokugamage and Pathberiya 2017) The term ‘Obstetric violence’ was defined in 2010 as “the appropriation of the body and reproductive processes of women by health personnel, which is expressed as dehumanized treatment, an abuse of medication, and to convert the natural processes into pathological ones, bringing with it loss of autonomy and the ability to decide freely about their bodies and sexuality, negatively impacting the quality of life of women” (Pérez D’gregorio Rogelio 2010).

There is a delicate balance of decolonising the body in maternity care which requires weighing the elements of obstetric interventions versus the ecological psychobiology (physiology) of birth. The consideration of decolonising the partogram, the most ubiquitous tool of obstetrics, does need to be raised and explored in the future.

#### Reverse innovation and cultural appropriation

This last section circles back to *epistemic bias* to provide insight into how women circumvent medicalised methods of maternity care and seek out methods to improve their wellbeing during pregnancy, childbirth and in the postnatal period that lie beyond the biomedical paradigm. *Reverse innovation*, which in itself has a controversial etymology, refers to the application of innovations from the Global South to problems in the Global North (Syed et al. 2013). Criticisms arise from the assumption of an established trend of innovation from the Global North to South, but nevertheless the paradigm shift it represents remains essential to decolonisation debate. Indigenous/traditional techniques which are commonly practised in Euro-America but originate in the Global South such as yoga, mindfulness and reflexology,(Tiran 2006; Shewamene et al. 2020) as well as Western medicines which are derived from traditional medicines(Maridass et al. 2008; Jones 2011) can be considered *reverse innovations*. While these practices provide benefit to mothers and have the potential to challenge the notion that the Global North is the centre of healthcare innovation, it also poses the risk of *cultural appropriation* if only tacit acknowledgement of an innovation’s origins is given. *Cultural appropriation* involves “*aspects of an oppressed culture being taken out of context by a historically dominant people, who lack the cultural context to properly understand, respect, or utilise these elements*”.(UCL Medical School n.d.) Therefore, reverse innovation is a double-edged sword; it has the potential to erode or reinforce the belief that the Global North is the epicentre of valid knowledge.

When proposing *reverse innovation*, it is, thus, important to consider the concepts of *cultural appropriation* and *cultural respect* if these interventions are to be incorporated into mainstream practice. Outside of routine medical practice, there are heated debates amongst allied maternity groups such as doulas or people of a minority ethnic origin, around the *cultural appropriation*^30^ of instructions on teaching to comfort and support mothers with modalities such as acupuncture, mindfulness, yoga and babywearing. There is some evidence demonstrating the benefits of these indigenous/traditional modalities of treatments. Studies show that acupuncture could relieve musculoskeletal pain,(Liddle and Pennick 2015) aid in cervical ripening(Smith et al. 2017), fetal positioning,(van den Berg et al. 2008; Li et al. 2009) improve rates of physiological births and women’s experience.(A. U. Lokugamage et al. 2020a, b) Mindfulness has been shown to improve mental health outcomes during the perinatal period.(Dhillon et al. 2017) Yoga can improve anxiety during pregnancy.(Newham et al. 2014) It is interesting that skin to skin contact which has demonstrable benefits to the baby(Moore et al. 2016) was originally a traditional technique from women of the Amazon basin and Andes of South America, for enhancing newborn survival but has been incorporated into mainstream biomedicine through an appropriation of the technique in a neonatal unit in Bogota, Columbia and is badged *‘Kangaroo care’*.^31^ Kangaroo care as a reverse innovation should acknowledge the indigenous/traditional roots of the intervention as a decolonial redressment.(Stefani et al. 2022).

Some of the above therapies come under the umbrella of complementary and alternative medicine (CAM) and the RCM position statement on these therapeutic modalities says that it has been estimated that as many as 87% of women use complementary therapies and/or natural remedies during pregnancy, childbirth and postnatally.(RCM 2017) This indicates that they are extremely popular amongst women. The RCM reports that many midwives are currently using some form of complementary therapy in their practice. However, channels for *reverse innovation* in maternity care may be stymied through UK clinical commissioning groups due to the advent of the recent *Evidence Based Interventions and Clinical Standards* (EBICS) policy(North London Partners in Health and Care 2019) which attempts to prevent mothers from accessing forms of CAM like acupuncture, yoga and mindfulness under the NHS. This is because mothers will not be funded unless there is a NICE (National Institute for Health and Care Excellence) recommendation. The EBICS policy does not seem to consider other well-recognised collections of evidence such as the Cochrane Reviews, adding to its already restrictive nature. This removes the opportunity for mainstream maternity care to incorporate *medical pluralism*, which is part of the decolonisation movement’s attempt to disrupt epistemic medical hegemony of healthcare. Although such therapies can be accessed by paying for them, this automatically creates inequality for women who cannot afford to pay for them. This move can even be seen as counterproductive to the vision of the *Better Births Initiative* (The National Maternity Review 2016), which outlines the importance of personalised care and listening to what is important for women. The EBICS policy is a top-down rationing initiative which may clash with the bottom-up approach of personalised care, hence, an area of decolonising tension. Returning back to our discussion point about women’s, autonomy and power imbalances, it will remain to be seen how this tension will play out over time.

We have summarised our analysis in Results Table 2:

**Table 2:** Themes discovered by an application of a decolonial lens to respectful maternity care in the UK

Analysis of professional structures and institutional biases revealed
1. An androcentric bias in obstetrics and gynaecology 2. The colonial legacy within higher education institutions 3. The need to decolonise medical and midwifery training across undergraduate and postgraduate teaching 4. Epistemic and geographical bias in knowledge production 5. Power imbalances in evidence-based medicine
Analysis of power imbalances between types of staff and stakeholders of care revealed
1. A long history of power imbalances between obstetrics and midwifery 2. A history of unconsented dehumanised experimentation on women to establish anatomical and physiological knowledge for obstetrics 3. Increased activity around redistribution of power to stakeholders and re-centring of the voices of women and birthing people
Analysis of person-centred care revealed
1. How women’s voices are being engaged in maternity services 2. The relevance of intersectionality regarding race and sexuality in maternity care 3. The need for Cultural Safety 4. Humanising the mechanical model of the biological female body 5. The value and complexity of reverse innovation 6. Necessity for greater awareness of cultural appropriation and respectful behaviours around it

## Discussion

### Limitations and strengths of this review

It has to be acknowledged that in the attempt to categorise, codify and striate the phenomena we have observed within our analysis and in formulating the table above, we risk ‘shoe-horning’ discourses into a Euro-American style academic hierarchy rather than accepting the spontaneity, fluidity and non-linearity of the bottom-up (people’s narrative-based) development of this knowledge. This juxtaposition of epistemologies is echoed in our interpretation of the obstetric versus midwifery paradigms of parturition in ensuing sections of this paper.

This review is a novel area of inquiry. The majority of our grey literature regarding public discourse and discussions were generated from engagement events in London and as such it is possible that our ideas are city centric and may not have captured local issues elsewhere in the UK, especially in the nations colonised by England (Scotland, Ireland, Wales). Nevertheless, social media, especially in the wake of the COVID-19 pandemic, has enabled connectivity and steady conversation amongst affected groups and with that we have foregrounded the dominant discourse. As mentioned in the introduction, we have not fully addressed the unresolved issue around nomenclature on which further scholarly inquiry surely will unravel.

Strengths of this decolonial paper include the diversity of the authors who have originated or lived or worked in the British Commonwealth. In addition, the authors’ collective expertise or publication record include decolonising higher education, social accountability in medical education, anthropology, epidemiology, power hierarchies in knowledge production, human rights in childbirth, obstetrics and physiological birth.

## Conclusion

We have discussed three generalised areas of power imbalance in maternity care:

I. Professional structures and institutional biases.
II. Power imbalances between types of staff and stakeholders of care.
III. Person-centred care through a decolonial lens

Highlighting these areas are necessary to address lingering structural biases and discrimination from colonial times that lead to institutionalised, professionalised and medicalised intersectional bias/oppression in the maternity area. In order to flatten power hierarchies, organisations, staff and service users need to amplify their knowledge of maternity care history and reflexivity around this subject matter as well as increase co-production of maternity services between stakeholders. There is evident overlap and interaction between these three arenas, and we must be flexible in negotiating between the interests of various stakeholders. Effective evaluation of existing norms will require intellectual humility and active listening by all providers of care, and for medical practitioners in particular to be conscious of their own professional biases, including the phenomena known as *‘white-coat privilege’*, or *‘white-coat fragility’*(A. U. Lokugamage et al. 2020b) *or epistemic fragility*.(Skopec et al. 2021) This may lead to a dangerous, defensive denial of the existence of structural bias and enduring enculturation within a professional body that has been, until recently, predominantly white, male and Eurocentric. Safety in healthcare is not just about discussion around mortality and morbidity. Cultural Safety teaches the point that people who have been affected by lingering colonial injustice need to feel safe and free from oppression when working or utilising maternity services.

Our results (Table 2) offer a tentative scaffold to examine maternity service in the UK through a decolonial angle, but we must be also mindful that ability to have the ‘headspace’ to learn, reflect and offer compassionate care is dependent on the working and learning conditions of the institutional environment. There is evidence that both obstetricians (Bourne et al. 2019) and midwives(Hunter et al. 2019) are experiencing ‘burn out’ and healthcare professionals in general are suffering ‘moral injury’^32^ from the toll the pandemic has taken in exacerbating existing gaps in NHS resources. When considering the present situation of the staff workforce the RCOG President Edward Morris has said.

“All recent national reports have identified that staff struggle with a lack of resources and capacity to provide best care. Excellent services mean staff are empowered to work to the best of their abilities in a system that values and supports them, in order to provide the best possible care for women and their families.”(RCOG 2020)

While we have examined maternity care services in the UK, other countries with different maternity care infrastructures and colonial histories will benefit from extrapolation of some of these concepts, to produce a decolonial framework that is appropriate to their particular healthcare setup. The issues we have raised serve as stepping stones to provide an impetus for future forums to emerge and is by no means the ‘be-all and end-all’ of this discourse. In order to achieve respectful maternity care, it is vital that contemporary maternity institutions understand the essential role that the decolonial perspective must play in uprooting historically entrenched inequity and provide support to ground-up initiatives by all stakeholders to bring about this change

## Data Availability

Not applicable.

## Footnotes

1 UCL. Dismantling the Master’s House [Internet]. University College London. [cited 2020 Apr 4]. Available from: http://www.dtmh.ucl.ac.uk/

2 Case law: *Montgomery v Lanarkshire Health Board* [2015] UKSC 11.

3 University of Glasgow—University news—Historic agreement sets up Glasgow-Caribbean Centre for Development Research [Internet]. [cited 2020 Apr 10]. Available from: https://www.gla.ac.uk/news/headline_667960_en.html

4 UCL. UCL announces action to acknowledge and address historical links with eugenics [Internet]. University College London. 2020 [cited 2020 Apr 10]. Available from: https://www.ucl.ac.uk/news/2020/feb/ucl-announces-action-acknowledge-and-address-historical-links-eugenics

5 SOAS. Decolonising Research Initiative Applying a Decolonial Lens to Research Development Practices [Internet]. SOAS University of London. [cited 2020 Apr 10]. Available from: https://www.soas.ac.uk/research/researchstrategy/decolonising-research-initiative/

6 BBC News. General election 2019: Labour plans to teach British Empire injustice in schools [Internet]. BBC. 2019 [cited 2020 Jan 26]. Available from: https://www.bbc.co.uk/news/election-2019-50551765

7 NHS. Vaginal mesh surgery should be a last resort, says NICE [Internet]. 2018 [cited 2020 Nov 5]. Available from: https://www.nhs.uk/news/medical-practice/vaginal-mesh-surgery-should-be-last-resort-says-nice/

8 UCL. Decolonising the Medical Curriculum [Internet]. University College London. [cited 2020 Apr 10]. Available from: https://www.ucl.ac.uk/culture/whats-on/decolonising-medical-curriculum

9 The Student Midwife—October 2020 | All4Maternity. https://www.all4maternity.com/ [Internet]. [cited 2020 Nov 5]; Available from: https://www.all4maternity.com/student-midwife-journal/student-midwife-october-2020/

10 Imperial College London. Examining geographic bias in our curricula [Internet]. Imperial College London. [cited 2020 Apr 10]. Available from: https://www.imperial.ac.uk/staff/educational-development/workshops/focus-on/geographic-bias/

11 Wickham S. Celebrating the Albany Practice Outcomes [Internet]. 2017 [cited 2020 Nov 5]. Available from: https://www.sarawickham.com/research-updates/celebrating-the-albany-practice-outcomes/

12 Maypilama Ḻäwurrpa, Josse P. Djäkamirr: Caretaker of Pregnancy and Birth [Internet]. One20 Productions; Molly Wardaguga Research Centre; Charles Darwin University; Northern Institute; 2021 [cited 2021 Jul 4]. Available from: https://www.birthingoncountry.com/djakamirr

13 BBC. Black and British: A Forgotten History, The Homecoming, The “Windrush” midwives [Internet]. BBC Two; 2016 [cited 2020 Nov 5]. Available from: https://www.bbc.co.uk/programmes/p04jm4pl

14 The RCOG welcomes the RCM re-location to London Bridge [Internet]. [cited 2020 Apr 10]. Available from: https://www.rcog.org.uk/en/news/rcog-welcomes-the-rcm-re-location/

15 National Maternity Voices [Internet]. [cited 2021 Feb 23]. Available from: http://nationalmaternityvoices.org.uk/

16 RCOG. RCOG Women’s Network [Internet]. The Royal College of Obstetricians and Gynaecologist. [cited 2020 May 15]. Available from: https://www.rcog.org.uk/en/patients/rcog-womens-network/

17 RCOG and RCM respond to UKOSS study of more than 400 pregnant women hospitalised with coronavirus [Internet]. [cited 2020 May 15]. Available from: https://www.rcog.org.uk/en/news/rcog-and-rcm-respond-to-ukoss-study-of-more-than-400-pregnant-women-hospitalised-with-coronavirus/

18 RCOG. International Women’s Day 2020: We need to talk about race. In: Royal College of Obstetricians and Gynaecologists [Internet]. 2020 [cited 2020 Apr 10]. Available from: https://www.rcog.org.uk/en/news/campaigns-and-opinions/international-womens-day/international-womens-day-2020/

19 RCOG and Five X More launch joint campaign to tackle racial disparities in maternity care [Internet]. [cited 2020 Nov 5]. Available from: https://www.rcog.org.uk/en/news/rcog-and-five-x-more-launch-joint-campaign-to-tackle-racial-disparities-in-maternity-care/

20 http://i3-uat.nz/our-work/projects/projects-library/care-standards/

21 https://www.nzno.org.nz/resources/whnau_ora

22 https://www.midwife.org.nz/wp-content/uploads/2019/02/Submission_WHAN%C4%80UORA_AUG2018.pdf

23 Godfrey-Isaacs L. What’s in a Name – Gender Inclusion in Maternity and Beyond [Internet]. All4Maternity. 2018 [cited 2020 Nov 5]. Available from: https://www.all4maternity.com/whats-in-a-name-gender-inclusion-in-maternity-and-beyond/ and. Brady M. LGBT health [Internet]. NHS England. [cited 2020 Nov 5]. Available from: https://www.england.nhs.uk/about/equality/lgbt-health/

24 See also Han E, O’Mahoney J. The British colonial origins of anti-gay laws. The Washington Post [Internet]. 2014 Oct 30 [cited 2021 Feb 23]; Available from: https://www.washingtonpost.com/news/monkey-cage/wp/2014/10/30/the-british-colonial-origins-of-anti-gay-laws/

25 Hunte B. Brighton NHS Trust introduces new trans-friendly terms [Internet]. BBC News. 2021. Available from: https://www.bbc.co.uk/news/uk-england-sussex-56007728

26 https://www.rcog.org.uk/news/draft-guideline-on-the-care-of-trans-and-gender-diverse-people-within-obstetrics-and-gynaecology-opens-for-consultation/

27 Gaskin IM. Going Backwards: The Concept of Pasmo [Internet]. 2003 [cited 2020 Nov 5]. Available from: https://inamay.com/going-backwards-the-concept-of-pasmo/

28 Wickham S. Stepping Stones and Cervical Wisdom [Internet]. 2019 [cited 2020 Nov 5]. Available from: https://www.sarawickham.com/articles-2/cervical-wisdom/

29 Davis B. JK. GI. The MANA Curve – Describing plateaus in labour using the MANA database. 2002;

30 Deshpande R. Difference Between Yoga Cultural Appropriation and Cultural Appreciation [Internet]. Yoga Journal. 2019 [cited 2020 May 23]. Available from: https://www.yogajournal.com/yoga-101/yoga-cultural-appropriation-appreciation; Not Your Idea: Cultural Appropriation in the Birthing Community—The Toast [Internet]. [cited 2020 May 15]. Available from: https://the-toast.net/2014/11/17/cultural-appropriation-birthing-community/ 123; and. White People are Commodifying Chinese Medicine Just Like They Did With Yoga [Internet]. [cited 2020 May 23]. Available from: https://wearyourvoicemag.com/white-people-chinese-medicine/

31 Personal communication from Dr Francoise Barbira Freedman (PhD) medical anthropologist, Cambridge, UK, who did extensive anthropological field work in the Amazon basin and Andes. *Kangaroo care* was not just an 'indigenous practice' in the Amazon and the Andes (a more extreme version of the 'normal' baby-carrying close to mother's chest-heart in the early months for babies born small or early). Until recently it has been the practice of choice to care for 'sietemesinos', babies born in the 7th and 8th months of gestation among urban mixed populations in the region.

32 BMA. Moral distress and moral injury: Recognising and tackling it for UK doctors. London; 2021.

## References

[CR1] Albers LL (1999). The duration of labor in healthy women. J Perinatol.

[CR2] Albers LL, Schiff M, Gorwoda JG (1996). The length of active labor in normal pregnancies. Obstet Gynecol.

[CR3] Angley, Meghan, Carla Clark, Renata Howland, Hannah Searing, Wendy Ma, Sang Hee Wilcox, Mph Won, et al. 2016. “Severe Maternal Morbidity in New York City, 2008–2012.” New York, NY.

[CR4] Asefa A (2021). Unveiling respectful maternity care as a way to address global inequities in maternal health. BMJ Glob Health.

[CR5] Baer, H.A., M. Singer, and I. Susser. 2003. *Medical Anthropology and the World System*. *Medical Anthropology and the World System*. Wrestport: Praeger.

[CR47] Bidwell G-U, Sevdalis N, Silverton L, Novis V, Freeman R, Hellyer A, Meulen J, Thakar R (2020). Impact of a quality improvement project to reduce the rate of obstetric anal sphincter injury: a multicentre study with a stepped-wedge design. BJOG Int J Obstet Gynaecol.

[CR7] Birthrights. 2022. “Systemic Racism, Not Broken Bodies An Inquiry into Racial Injustice and Human Rights in UK Maternity Care.” London.

[CR8] Bougie O, Yap MI, Sikora L, Flaxman T, Singh S (2019). Influence of race/ethnicity on prevalence and presentation of endometriosis: a systematic review and meta-analysis. BJOG: Int J Obstet Gy.

[CR9] Bourne T, Shah H, Falconieri N, DIrk Timmerman, Christoph Lees, Alison Wright, Mary Ann Lumsden, Lesley Regan, and Ben Van Calster.  (2019). Burnout, well-being and defensive medical practice among obstetricians and gynaecologists in the UK: cross-sectional survey study. BMJ Open.

[CR10] Boutet G (2012). Breast inflammation: clinical examination, aetiological pointers. Diagn Interv Imaging.

[CR11] British Association of Dermatologists. 2022a. “Researchers Propose New Evidence-Based Approach to Describing Skin Colour.” *British Association of Dermatologists*, 2022a.

[CR12] British Association of Dermatologists. 2022b. “Skin of Colour Education in the UK.” British Associaation of Dermatologiss. 2022b. https://www.bad.org.uk/education-training/skin-of-colour-in-dermatology-education/?fbclid=IwAR05LVMn2zwsitgh-oQdrHJ0PPSUMKnKjbAWuzrxJ8zqD0uPK2DxUBhuxbo.

[CR13] Buckley SJ (2015). Hormonal physiology of childbearing: evidence and implications for women, babies, and maternity care. J Perinat Educ.

[CR14] Buckley, Sarah, and Uvnäs Moberg K. n.d. “Nature and consequences of of oxytocin and other neurohormones during pregnancy and childbirth. In: Squaring the Circle: Researching Normal Childbirth in a Technologicl World. .”

[CR15] Cahill HA (2001). Male appropriation and medicalization of childbirth: an historical analysis. J Adv Nurs.

[CR16] Cerdeña JP, Plaisime MV, Tsai J (2020). From race-based to race-conscious medicine: how anti-racist uprisings call us to act. The Lancet.

[CR17] Chimakonam J, du Toit L (2018). African philosophy and the epistemic marginalization of women.

[CR33] Christine D, Lokugamage AU (2021). Racial profiling for induction of labour: improving safety or perpetuating racism?. BMJ.

[CR49] Claire H (2014). Exploring same sex couples’ experiences of maternity care. Br J Midwifery.

[CR18] Chow H, Lokugamage AU, Gishen F (2019). Diversity health checks in undergraduate curricula. Clin Teacher.

[CR19] Columbia Law School. 2017. “Kimberlé Crenshaw on Intersectionality, More than Two Decades Later.” Columbia Law School. 2017. https://www.law.columbia.edu/news/archive/kimberle-crenshaw-intersectionality-more-two-decades-later.

[CR20] Cordeiro-Rodrigues L (2020). Toward a decolonized healthcare ethics: colonial legacies and the siamese crocodile. Dev World Bioeth.

[CR21] Cormier JN, Xing Y, Ding M, Lee JE, Mansfield PF, Gershenwald JE, Ross MI, Xianglin LDu (2006). Ethnic Differences among patients with cutaneous melanoma. Arch Intern Med.

[CR22] Cumberlege J (2020). First do no harm: the report of the independent medicines and medical devices safety review.

[CR23] Dahlen HG, Drandic D, Shah N, Cadee F, Malata A (2022). Supporting midwifery is the answer to the wicked problems in maternity care. Lancet Glob Health.

[CR24] Dahlen HG, Thornton C, Downe S, De Jonge A, Seijmonsbergen-Schermers A, Tracy S, Tracy M, Bisits A, Peters L (2021). Intrapartum interventions and outcomes for women and children following induction of labour at term in uncomplicated pregnancies: a 16-year population-based linked data study. BMJ Open.

[CR25] Davis G, Waldman B, Phipps H, Hyett J, Vries B (2020). A survey of obstetricians’ attitudes to induction of labour at 39 weeks gestation with the intention of reducing caesarean section rates. Australian New Zealand J Obstet Gynaecol.

[CR26] Davis-Floyd R, Pascali-Bonaro D, Leslie MS, Ponce RG, de León.  (2011). The international motherbaby childbirth initiative: working to create optimal maternity care worldwide. Int J Childbirth.

[CR27] DeAngelis, Tori. 2002. “A New Generation of Issues for LGBT Clients.” *American Psychological Association* 33 (2): undefined.

[CR28] Deery R (2003). Engaging with clinical supervision in a community midwifery setting: an action research study.

[CR29] Department of Health and Social Care. 2022. “Ockenden Report.” London.

[CR30] Department of Reproductive Health and Research, World Health Organization. 2017. “Prevention and Elimination of Disrespect and Abuse during Childbirth.” *WHO*. Geneva: World Health Organization.

[CR31] Dhillon A, Sparkes E, Duarte RV (2017). Mindfulness-based interventions during pregnancy: a systematic review and meta-analysis. Mindfulness.

[CR32] Diangelo Robin. 2011. White Fragility. *International Journal of Critical Pedagogy*. 3.

[CR34] Downes, Heidi. 2020. “Honouring the Slaves Experimented on by the ‘Father of Gynaecology.’” The Conversation. 2020. https://theconversation.com/honouring-the-slaves-experimented-on-by-the-father-of-gynaecology-148273.

[CR35] Draper, Elizabeth S, Ian D Gallimore, Jennifer J Kurinczuk, Sara Kenyon, and MBRRACE-UK. 2021. “MBRRACE-UK Perinatal Confidential Enquiry: Stillbirths and Neonatal Deaths in Twin Pregnant.” Leicester.

[CR36] Dundes L (1987). Public health then and now the evolution of maternal birthing position. Am J Public Health.

[CR37] Ehrenreich B, English D (2010). Witches, midwives & nurses: a history of women healers. Witches Midwives Nurses.

[CR126] Eve T, Wayne Yang K (2012). Decolonization is not a metaphor. Decolonization Indigeneity Edu Soc.

[CR39] Francis R (2013). Report of the mid staffordshire NHS foundation trust public inquiry. New Dir Youth Dev.

[CR40] FRIEDMAN, E.  (1954). The graphic analysis of labor. Am J Obstet Gynecol.

[CR41] Gaskin, Ina May. 2003. “Going Backwards: The Concept of Pasmo.” 2003. https://inamay.com/going-backwards-the-concept-of-pasmo/. 14533272

[CR42] Ghui R, Bansal JK, McLaughlin C, Kotaska A, Lokugamage AU (2016). An evaluation of the guidelines of the society of obstetricians and gynaecologists of Canada. J Obstet Gynaecol.

[CR43] GMC. 2018. “Progression Reports.” The General Medical Council. 2018. https://www.gmc-uk.org/education/reports-and-reviews/progression-reports.

[CR44] Gribble KD, Bewley S, Bartick MC, Mathisen R, Walker S, Gamble J, Bergman NJ, Gupta A, Hocking JJ, Dahlen HG (2022). Effective communication about pregnancy, birth, lactation, breastfeeding and newborn care: the importance of sexed language. Front Global Women’s Health.

[CR45] Grobman WA, Rice MM, Reddy UM, Tita ATN, Silver RM, Mallett G, Hill K (2018). Labor induction versus expectant management in low-risk nulliparous women. N Engl J Med.

[CR46] Groeschel N, Glover P (2001). The partograph. Used daily but rarely questioned. Australian J Midwifery.

[CR48] Hamberg K (2008). Gender bias in medicine. Women’s Health.

[CR50] Han E, O’Mahoney J (2014). British colonialism and the criminalization of homosexuality. Camb Rev Int Aff.

[CR51] Haskell H (2020). Cumberlege review exposes stubborn and dangerous flaws in healthcare. The BMJ.

[CR52] Hidalgo DC, Olusanya O, Harlan E (2021). Critical care trainees call for pulse oximetry reform. Lancet Respir Med.

[CR53] Holdcroft A (2007). Gender bias in research: how does it affect evidence based medicine?. J R Soc Med.

[CR54] Hunter B, Fenwick J, Sidebotham DM, Henley DJ (2019). Midwives in the United Kingdom: levels of burnout, depression, anxiety and stress and associated predictors. Midwifery.

[CR55] Hussain-Gambles M, Atkin K, Leese B (2004). Why ethnic minority groups are under-represented in clinical trials: a review of the literature. Health Soc Care Community.

[CR56] Jackson G (2019). Pain and prejudice: a call to arms for women and their bodies.

[CR57] JERVIS, BK.  (2019). An ethnographic exploration of womens midwives and obstetricians beliefs about maternal movement during labour.

[CR58] Joint Royal College of Physicians Training Board. 2018. “2017 Survey of Medical Certificate of Completion of Training (CCT) Holders’ Career Progression.” Joint Royal College of Physicians. 2018. https://www.rcplondon.ac.uk/projects/outputs/2017-survey-medical-certificate-completion-training-cct-holders-career-progression.

[CR129] Joeri V, Luyben A, O’Connell R, Gillen P, Escuriet R, Fleming V (2019). Failure or progress?: the current state of the professionalisation of midwifery in Europe. Eur J Midwifery.

[CR59] Jones AW (2011). Early drug discovery and the rise of pharmaceutical chemistry. Drug Test Anal.

[CR38] Kazeem F, Adeyelure M (2016). Decolonizing bioethics in African. Beonline.

[CR60] Kirkup, Bill. 2015. *The Report of the Morecambe Bay Investigation*.

[CR61] Kmietowicz, Zosia. 2020. “Racism in Medicine (Special Edition).” The BMJ. 2020. https://www.bmj.com/racism-in-medicine.

[CR62] Knight, Marian, Kathryn Bunch, Derek Tuffnell, Hemali Jayakody, Judy Shakespeare, Rohit Kotnis, Sara Kenyon, and Jennifer J Kurinczuk. 2018. “Saving Lives, Improving Mothers’ Care - Lessons Learned to Inform Maternity Care from the UK and Ireland Confidential Enquiries into Maternal Deaths and Morbidity 2014–16.” Oxford.

[CR63] Kymlicka W (2018). Connecting domination contracts. Ethn Racial Stud.

[CR64] Lalonde A, Herschderfer K, Pascali-Bonaro D, Hanson C, Fuchtner C, Visser GHA (2019). The international childbirth initiative: 12 steps to safe and respectful motherbaby–family maternity care. Int J Gynecol Obstet.

[CR65] Lavender T, Cuthbert A, Smyth RMD (2018). Effect of partograph use on outcomes for women in spontaneous labour at term and their babies. Cochrane Database Syst Rev.

[CR66] Laverty M, McDermott DR, Calma T (2017). Embedding cultural safety in Australia’s main health care standards. Med J Aust.

[CR67] Lempp H, Seale C (2004). The hidden curriculum in undergraduate medical education: qualitative study of medical students’ perceptions of teaching. BMJ.

[CR68] Leonardo Z, Porter RK (2010). Pedagogy of fear: toward a fanonian theory of ‘safety’ in race dialogue. Race Ethn Educ.

[CR69] LGBT Foundation. 2022. “Trans + Non Binary Experiences of Maternity Services - Survey Findings, Report and Recommendations.”

[CR70] Li X, Jun Hu, Wang X, Zhang H, Liu J (2009). Moxibustion and other acupuncture point stimulation methods to treat breech presentation: a systematic review of clinical trials. Chinese Medicine.

[CR71] Liddle SD, Pennick V (2015). Interventions for preventing and treating low-back and pelvic pain during pregnancy. Cochrane Database Syst Rev.

[CR72] Lokugamage AU, Eftime VAI, Porter D, Ahillan T, Ke SX (2020). Birth preparation acupuncture for normalising birth: an analysis of NHS service routine data and proof of concept. J Obstet Gynaecol.

[CR73] Lokugamage AU, Pathberiya SDC (2017). Human rights in childbirth, narratives and restorative justice: a review. Reprod Health.

[CR74] Lokugamage, A. U., and Alice Meredith. 2020. “Women from Ethnic Minorities Face Endemic Structural Racism When Seeking and Accessing Healthcare - The BMJ.” BMJ. 2020. https://blogs.bmj.com/bmj/2020/03/05/women-from-ethnic-minorities-face-endemic-structural-racism-when-seeking-and-accessing-healthcare/.

[CR75] Lokugamage AU, Ahillan T, Pathberiya SDC (2020). Decolonising ideas of healing in medical education. J Med Ethics.

[CR76] Lokugamage AU, Wong SHM, Robinson NMA, Pathberiya SDC (2021). Transformational learning to decolonise global health. The Lancet.

[CR77] Lokugamage AU, Rix E, Fleming T, Meredith A, Khetan T, Hastie C (2021). Translating cultural safety to the UK. J Med Ethics.

[CR78] Lorde, Audre. 2007. “The Master’s Tools Will Never Dismantle the Master’s House.” In *Sister Outsider: Essays and Speeches*, 110–14. Berkeley, CA: Crossing Press.

[CR79] Machin D, Scamell M (1997). The experience of labour: using ethnography to explore the irresistible nature of the bio-medical metaphor during labour. Midwifery.

[CR80] Manning, Sarah Sunshine. 2018. “Decolonizing Birth.” Open Democracy. 2018. https://www.opendemocracy.net/en/transformation/decolonizing-birth/.

[CR81] Maridass M, De John A, Britto.  (2008). Origins of plant derived medicines. Ethnobotanical Leaflets..

[CR82] Marland H (1994). The art of midwifery: early modern midwives in europe (wellcome institute series in the history of medicine).

[CR83] Matias CE (2016). Feeling white: whiteness, emotionality, and education (cultural pluralism, democracy, socio-environmental justice & education).

[CR84] Matias CE, Mackey J (2016). Breakin’ down whiteness in antiracist teaching: introducing critical whiteness pedagogy. Urban Review.

[CR85] Moore ER, Bergman N, Anderson GC, Medley N (2016). Early skin-to-skin contact for mothers and their healthy newborn infants.

[CR86] Murphy-Lawless J (1998). Reading birth and death: a history of obstetric thinking.

[CR87] Nash JC (2008). Re-thinking intersectionality. Fem Rev.

[CR88] Newham JJ, Wittkowski A, Hurley J, Aplin JD, Westwood M (2014). Effects of antenatal yoga on maternal anxiety and depression: a randomized controlled trial. Depress Anxiety.

[CR89] NHS digital service manual. 2020. “Content Style Guide - Inclusive Language.” NHS. 2020. https://service-manual.nhs.uk/content/inclusive-language.

[CR90] NHS Improvement. 2018. “Patient Experience Improvement Framework | NHS Improvement.” NHS. 2018. https://improvement.nhs.uk/resources/patient-experience-improvement-framework/.

[CR91] NHS National Quality Board (2012). NHS Patient Experience Framework.

[CR92] North London Partners in Health and Care. 2019. “Evidence Based Interventions and Clinical Standards 2019.” London.

[CR93] Opara, Ijeoma. 2021. “It’s Time to Decolonize the Decolonization Movement - Speaking of Medicine and Health.” PLOS. July 29, 2021. https://speakingofmedicine.plos.org/2021/07/29/its-time-to-decolonize-the-decolonization-movement/.

[CR94] Pan RK, Kaski K, Fortunato S (2012). World citation and collaboration networks: uncovering the role of geography in science. Sci Rep.

[CR95] Papps E, Ramsden I (1996). Cultural safety in nursing: the New Zealand Experience. Int J Qual Health Care.

[CR96] Pérez D’gregorio, Rogelio.  (2010). Obstetric violence: a new legal term introduced in Venezuela. Int J Gynecol Obstet.

[CR97] Petch, Michael. 2020. “New Research Project Launched Looking into the Experiences of Trans Men and Non-Binary People Using Maternity Services.” LGBT Foundation. 2020. https://lgbt.foundation/news/new-research-project-launched-looking-into-the-experiences-of-trans-men-and-non-binary-people-using-maternity-services/393?fbclid=IwAR2xHJf9_7w_fzEUwQxhrAF_l0bUXDQWewIDEvGTbnwKxE8cgd8-OUnYmYw.

[CR98] Peter, M;, and R Wheeler. 2022. “Black Maternal Experinces Report.” London.

[CR99] Philpott RH (1972). Graphic records in labour. BMJ.

[CR100] Philpott RH, Castle WM (1972). Cervicographs in the management of labour in primigravidae. I. The alert line for detecting abnormal labour. BJOG Int J Obstetrics Gynaecol.

[CR101] Prusova K, Churcher L, Tyler A, Lokugamage AU (2014). Royal college of obstetricians and gynaecologists guidelines: how evidence-based are they?. J Obstet Gynaecol.

[CR102] RCM. 2017. “Position Statement - Complementary Therapies and Natural Remedies.” London.

[CR103] RCOG. 2018. “O&G Workforce Report 2018 Update on Workforce Recommendations and Activities.” London.

[CR104] RCOG. 2020. “Actions in Donna Ockenden Review Must Be Acted upon Immediately by All Maternity Services Say RCOG and RCM.” Royal College of Obstetricians and Gynaecologists. December 10, 2020. https://www.rcog.org.uk/en/news/actions-in-donna-ockenden-review-must-be-acted-upon-immediately-by-all-maternity-services-say-rcog-and-rcm/.

[CR105] RCOG. 2022. “RCOG Essential Reading .” Royal College of Obstetricians and Gynaecologists. 2022. https://elearning.rcog.org.uk/differential-attainment/essential-reading.

[CR106] Rimmer A (2020). Presenting clinical features on darker skin: Five Minutes with … Malone Mukwende. BMJ.

[CR107] Royal College of Midwives, and Royal College of Obstetricians and Gynaecologists. 2020. “Guidance for Provision of Midwife-Led Settings and Home Birth in the Evolving Coronavirus (COVID-19) Pandemic -Information for Healthcare Professionals.” London.

[CR108] Sandall J, Soltani H, Gates S, Shennan A, Devane D (2016). Midwife-led continuity models versus other models of care for childbearing women. Cochrane Database Syst Rev.

[CR109] Rupal S, Ahluwalia S (2019). Editorials the challenges of understanding differential attainment in postgraduate medical education. Br J Gen Pract.

[CR110] Shewamene Z, Dune T, Smith CA (2020). Use of traditional and complementary medicine for maternal health and wellbeing by African migrant women in Australia: a mixed method study. BMC Complement Med Ther.

[CR111] Simons RL, Lei MK, Beach SRH, Philibert RA, Cutrona CE, Gibbons FX, Barr A (2016). Economic hardship and biological weathering: the epigenetics of aging in a U.S. sample of black women. Soc Sci Med.

[CR112] Skopec Mark, Molly Fyfe, Hamdi Issa, Kate Ippolito, Mark Anderson, and Matthew Harris. 2021. “Decolonization in a higher education STEMM Institution – Is ‘Epistemic Fragility’ a barrier?” *London Review of Education*. 10.14324/lre.19.1.18.

[CR113] Skopec M, Issa H, Reed J, Harris M (2020). The role of geographic bias in knowledge diffusion: a systematic review and narrative synthesis. Res Integrity Peer Rev.

[CR114] Smith CA, Armour M, Dahlen HG (2017). Acupuncture or acupressure for induction of labour. Cochrane Database Syst Rev.

[CR115] Spettel S, White MD (2011). The portrayal of J. Marion sims’ controversial surgical legacy. J Urol.

[CR116] Stefani G, Skopec M, Battersby C, Harris M (2022). Why is kangaroo mother care not yet scaled in the UK? a systematic review and realist synthesis of a frugal innovation for newborn care. BMJ Innovations.

[CR117] Studd J (1973). Partograms and nomograms of cervical dilatation in management of primigravid labour. BMJ.

[CR118] Sullivan S (2013). Inheriting racist disparities in health. Crit Philos Race.

[CR119] Syed SB, Dadwal V, Martin G (2013). Reverse innovation in global health systems: towards global innovation flow. Glob Health.

[CR120] Tahmasebi H, Trajcevski K, Higgins V, Adeli K (2018). Influence of ethnicity on population reference values for biochemical markers. Crit Rev Clin Lab Sci.

[CR121] The Glossary of Education Reform. 2015. “Hidden Curriculum Definition.” Great Schools Partnership . 2015. https://www.edglossary.org/hidden-curriculum/.

[CR122] The National Maternity Review. 2016. “BETTER BIRTHS Improving Outcomes of Maternity Services in England A Five Year Forward View for Maternity Care.”

[CR123] The WHO Reproductive Health Library (2018). WHO Recommendation on Respectful Maternity Care during Labour and Childbirth.

[CR124] Thornton JG, Dahlen HG (2020). The UK obstetric anal sphincter injury (OASI) care bundle: a critical review. Midwifery.

[CR125] Tiran D (2006). Complementary therapies in pregnancy: midwives’ and obstetricians’ appreciation of risk. Complement Ther Clin Pract.

[CR127] Turner MA, Kelly M, Leftwick P, Dogra N (2014). Tomorrow’s doctors and diversity issues in medical education. Med Teacher.

[CR128] UCL Medical School. n.d. “Connecting up the Curriculum – Decolonising the Medical Curriculum.” University College London. Accessed April 17, 2020. https://decolonisingthemedicalcurriculum.wordpress.com/connecting-up-the-curriculum/.

[CR6] van den Berg I, Bosch JL, Jacobs B, Bouman I, Duvekot JJ, Myriam Hunink MG (2008). Effectiveness of acupuncture-type interventions versus expectant management to correct breech presentation: a systematic review. Complement Ther Med.

[CR130] Walsh D (1994). Management of progress in the first stage of labour. Midwives Chron.

[CR131] Walsh D (2006). Subverting the assembly-line: childbirth in a free-standing birth centre. Soc Sci Med.

[CR132] Walsh DJ (2010). Childbirth embodiment: problematic aspects of current understandings. Sociol Health Illn.

[CR133] Walsh, Denis, and Mary Steen. 2007. “The Role of the Midwife: Time for a Review.” Midwives Magazine. July 2007. https://www.rcm.org.uk/news-views/rcm-opinion/the-role-of-the-midwife-time-for-a-review/. 17654848

[CR134] White, Nadine. 2020. “Black Women Were Tortured To Develop Gynaecology Methods. Midwives Want Them Remembered.” HuffPost UK. 2020. https://www.huffingtonpost.co.uk/entry/a-campaign-to-honour-the-unsung-black-mothers-of-gynaecology_uk_5f106cedc5b6d14c33644d56?guce_referrer=aHR0cHM6Ly93d3cuZ29vZ2xlLmNvbS8&guce_referrer_sig=AQAAAE_iBrpXArOhWpPi6WTY8tRPd8aCrPh939Eu5IC-m8QNdhEMUFcasaMKhVK.

[CR135] WHO. 2015. *Pregnancy, Childbirth, Postpartum and Newborn Care: A Guide for Essential Practice Third Edition*. 26561684

[CR136] Wong, Sarah Hui Min, Faye Gishen, and A. U. Lokugamage. 2021. Decolonising the medical curriculum‘: humanising medicine through epistemic pluralism, cultural safety and critical consciousness *London Review of Education*. 10.14324/LRE.19.1.16.

[CR137] Woolf K (2020). Differential attainment in medical education and training. The BMJ.

[CR138] Woolf K, Potts HWW, McManus IC (2011). Ethnicity and academic performance in UK trained doctors and medical students: systematic review and meta-analysis. BMJ.

[CR139] Woolf K, Rich A, Viney R, Needleman S, Griffin A, London E, Surrey K (2016) Perceived causes of differential attainment in UK postgraduate medical training: A national qualitative study. BMJ Open 6:e013429. 10.1136/bmjopen-2016-013429 10.1136/bmjopen-2016-013429 PMC5168507 27888178

[CR140] WRES Implementation team. 2021. “NHS Medical Workforce Race Equality Standard (MWRES): 2020 Data Analysis Report for the NHS Medical Workforce.”

[CR141] Wright JM (2007). Practice guidelines by specialist societies are surprisingly deficient. Int J Clin Pract.

[CR142] Wright JD, Pawar N, Gonzalez JSR, Lewin SN, Burke WM, Simpson LL, Charles AS, Mary E, DʼAlton, and Thomas J. Herzog.  (2011). Scientific evidence underlying the American College of obstetricians and gynecologistsʼ practice bulletins. Obstet Gynecol.

